# RNA Interference based Approach to Down Regulate Osmoregulators of Whitefly (*Bemisia tabaci*): Potential Technology for the Control of Whitefly

**DOI:** 10.1371/journal.pone.0153883

**Published:** 2016-04-22

**Authors:** Amir Raza, Hassan Jamil Malik, Muhammad Shafiq, Imran Amin, Jodi A. Scheffler, Brian E. Scheffler, Shahid Mansoor

**Affiliations:** 1 Molecular Virology and Gene Silencing Laboratory, Agricultural Biotechnology Division, National Institute for Biotechnology and Genetic Engineering (NIBGE), Jhang Road, PO Box # 577, Faisalabad, Pakistan; 2 Pakistan Institute of Engineering and Applied Sciences (PIEAS), Islamabad, Pakistan; 3 USDA-ARS, Crop Genetics Research Unit, 141 Experiment Station Rd, Stoneville, Mississippi, 38776, United States of America; 4 USDA-ARS, Genomics and Bioinformatics Research Unit, 141 Experiment Station Rd, Stoneville, Mississippi, 38776, United States of America; Kansas State University, UNITED STATES

## Abstract

Over the past decade RNA interference (RNAi) technology has emerged as a successful tool not only for functional genomics, but *in planta* expression of short interfering RNAs (siRNAs) that could offer great potential for insect pest management. The diet of insects feeding exclusively on phloem sieves contains water and sugars as main components, and the uptake of the liquid food greatly depends on the osmotic pressure within the insect body. Based on this physiological mechanism, transgenic plants of *Nicotiana tabacum* were generated expressing double stranded RNA (dsRNA) against both *aquaporin* (*AQP*) and a sucrase gene, *alpha glucosidase* (*AGLU*). These two genes are involved in osmotic pressure maintenance particularly in sap sucking insects, and the aim was to disrupt osmoregulation within the insect ultimately leading to mortality. Real time quantitative PCR (RT-qPCR) was performed to assess the suppression of gene expression in *Bemisia tabaci* (*B*. *tabaci*) and mortality was recorded during transgenic tobacco feeding bioassays. Feeding of insects on plants expressing dsRNA significantly reduced the transcript level of the target genes in *B*. *tabaci* after six days of feeding and more than 70% mortality was observed in *B*. *tabaci* fed on transgenic plants compared to the control plants. Our data shows that down-regulation of genes related to osmoregulation may find practical applications for the control of this important pest in cotton and other crops.

## Introduction

Extensive losses to agricultural produce worldwide are due to a number of biotic and abiotic stresses, and there is increased demand for transgenic crops with the ability to withstand these stresses. The exceptional success of insecticidal protein based Bt technology for the control of *dipteran* and *lepidopteran* insects of cotton, maize and canola has significantly reduced the use of insecticides. More than 200 toxic Bt proteins have been identified and isolated from different strains of *Bacillus thuringiensis* with a varying degree of toxicity [1]. Because of the extensive adoption of genetically modified crops expressing toxins against chewing insects another class of insects, known as sucking insects, which includes aphid, mealybug, jassid and whitefly have emerged as pests of serious concern [2, 3].

Previously considered a minor pest, *Bemisia tabaci* belonging to the order *Hemiptera*, sub-order *Sternorryncha* and family *Aleyrodidae* is an important insect pest of agricultural crops throughout the world. *B*. *tabaci* not only damages the plant by sucking vital sap from the phloem tissue but also transmits numerous plant virus species. It exclusively transmits more than 200 plant-infecting virus species [4] to a wide range of host plants. Genetic divergence of *B*. *tabaci* has been under investigation for a long time. The *Bemisia* species complex is considered as morphologically indistinguishable cryptic species [5] which shows phenotypic polymorphism, however the question was posed recently that: is it a single species complex having several biotypes or a complex of several species [6]. Phylogenetic studies based on mitochondrial cytochrome oxidase I (mtCOI) gene revealed that *B*. *tabaci* is a complex of multiple species which contains at least 34 putative species [7–10]. With the increase in monoculture production of crops, vegetables and most recently the increase in global trade of ornamental plants [10], *B*. *tabaci* has become one of the most important phloem feeding pests and virus vectors affecting economically important food, fiber, and nursery crops worldwide.

Use of insecticides for the control of insect pests is a major environmental as well as health concern and long term usage also leads to the development of insect resistance to these harsh chemicals, thereby worsening the problem. Development of resistance has been documented particularly for *B*. *tabaci* which is resistant to different chemical pesticides, including *pyriproxyfen* and *buprofezin* [11, 12], *imidacloprid* [13] etc. Therefore, technologies which limit the insect population on crop plants in an environment friendly manner and also provide long lasting solution to the problem are much needed.

Over the past decade, RNA interference technology has demonstrated the capacity as a successful tool for controlling the insect pests of important crops. RNAi is a gene silencing technology which makes use of double stranded RNA to interfere with the gene function directed against messenger RNA (mRNA) of specific gene target(s) or its promoter region [14]. The phenomenon is also known as post transcriptional gene silencing (PTGS) and there are three basic steps in the RNAi process [15, 16]. In the first step, the long dsRNA molecule is cleaved into short RNA partial dimers by a ribonuclease III enzyme called dicer. Double stranded RNA acts as a trigger for the silencing machinery whereas the dicer enzyme cleaves the dsRNA into short 21–25 nucleotide (nt) RNA molecules. The guide strand of short interfering RNAs (siRNAs) gets incorporated into the RNA induced silencing complex (RISC) and leads the complex to the target mRNA for degradation.

A number of RNAi studies performed on *B*. *tabaci* demonstrates the potential of this technology for the control of whitefly. Initially delivery of dsRNA using microinjection resulted in 70% decrease in the expression level of different genes in *Bemisia* [17] and later on another study used artificial diet based approach to deliver dsRNA orally against five different genes including RPL9 and V-ATPase A being the most significant targets resulting in more than 90% mortality of *B*. *tabaci* because of expression down regulation [18]. Plants can also be genetically engineered to induce an RNAi response against the insect pests [19]. *In planta* expression of dsRNA complementary to housekeeping or parasitism genes of root-knot nematode resulted in resistance against the nematode [20, 21]. The effect of RNAi varies from species to species and sometimes even within the same species of insects [22–24]. The reason could be that the gene selected as the target for RNAi has tissue specific expression in a region of the insect body not easily accessible to siRNAs produced. In most of the previously reported studies involving agriculturally important insect pests, RNAi was aimed at midgut or salivary glands of the insects assuming that the maximum dsRNA uptake occurs in these parts. Some of the examples include *cytochrome P450* gene in *Helicoverpa armigera* [25], *V-ATPase* gene in *Diabrotica virgifera virgifera* [26] and *aquaporin* and *C002* genes in *Acyrthosiphon pisum* [27, 28]. A recent study reported that transgenic plants expressing dsRNA against *v-ATPase A* gene of *B*. *tabaci* resulted not only in suppression of gene expression but also lead to significantly higher mortality and great decline in *Bemisia* population [29]. The study also demonstrated the prevention of other potential losses to plants including loss of sugar content from plants because of *Bemisia* feeding.

Expression down-regulation of specific genes using RNAi technology has been the method of choice for insect control and functional genomics research. Initially the technology relied on the use of microinjection and other artificial diet based feeding methods [24], which were inapplicable in the field conditions and new methods of dsRNA delivery needs to be explored. One approach is to identify the genes of interest and develop transgenic plants in order to assess the efficacy of the disruption of the target gene(s) to confer insect/pest or disease resistance. Double stranded RNA being expressed against specific gene targets either transiently or stably in plant systems can induce suppression of target genes. Genetic engineering based host plant mediated insect resistance has evolved as a viable method [30, 31]. Plant mediated genetically engineered resistance using a wide range of plant species including *Oryza sativa*, *Arabidopsis thaliana*, *Gossypium hirsutum*, *Nicotiana tabacum* has been demonstrated to be effective against herbivores belonging to different insect orders including *Coleoptera*, *Lepidoptera* and *Hemiptera* [19, 25, 26, 32, 33].

The diet of insects feeding exclusively on phloem sieves contains water and sugars as the main components, and the uptake of the liquid food depends on the osmotic pressure within the insect body. Based on this dependence on osmotic pressure, dsRNA was expressed against *aquaporin* (*AQP*) and a sucrase gene, *alpha glucosidase* (*AGLU*) with the aim to disrupt osmoregulation. This study was designed to evaluate if the expression of dsRNA simultaneously against two important whitefly genes could produce siRNAs sufficient to cause mortality of this sucking pest.

## Materials and Methods

### Insect rearing

*B*. *tabaci* insects were initially collected from the cotton fields of Nuclear Institute for Agriculture and Biology (NIAB), Faisalabad, Pakistan and were maintained on potted cotton plants at 7–10 leaf stages (*Gossypium hirsutum*) in insect proof cages under the controlled conditions at a temperature of 26 ±2°C and 60–70% relative humidity with a 16:8 h light and dark photoperiod. The *B*. *tabaci* colony was maintained in a separate room specified for insect rearing in insect free cages through serial transfer at four to six weeks intervals using a modified method described earlier [34].

### Construction of dsRNA expression cassette

**Fig 1:**
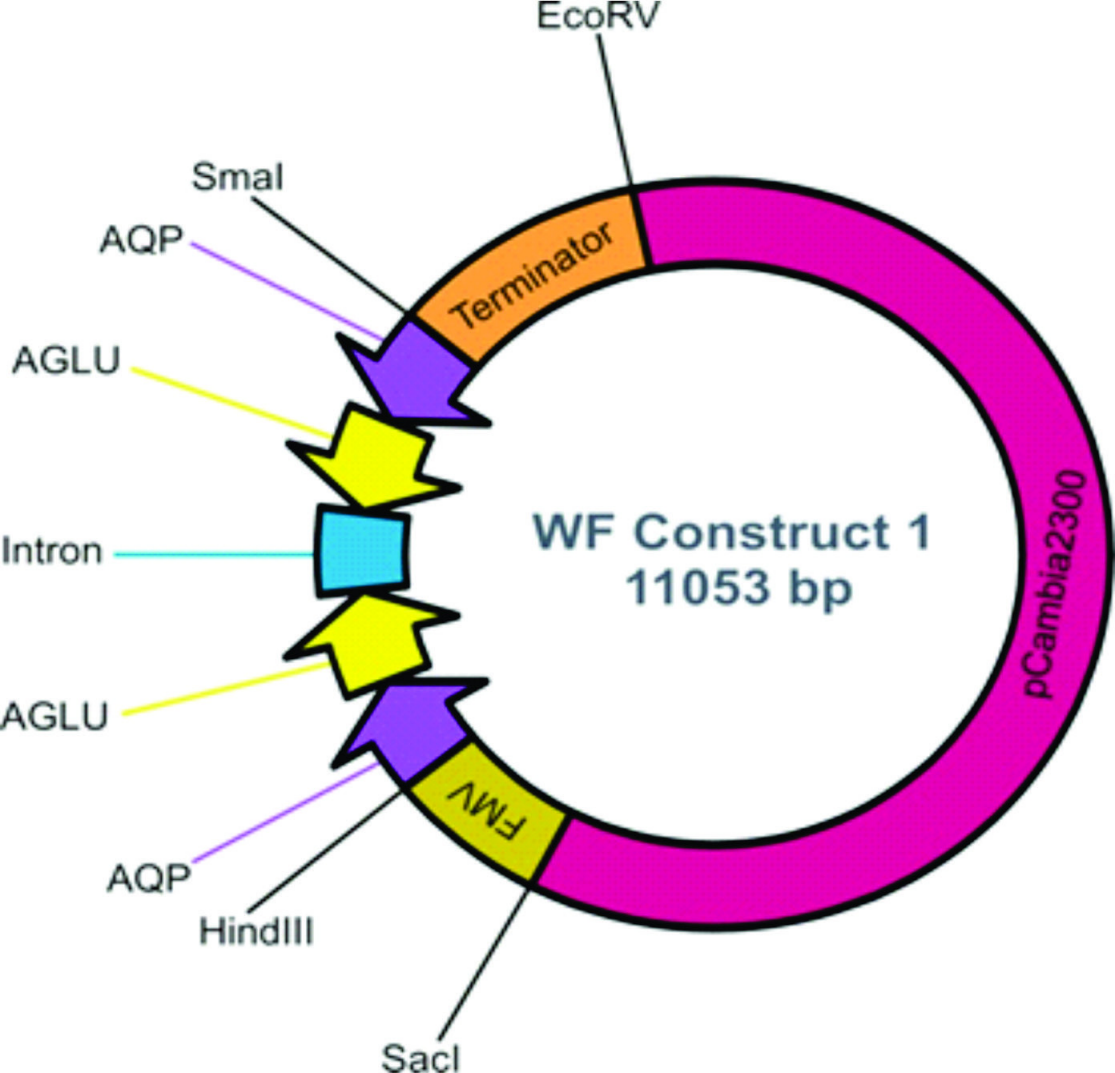
Construct for plant transformations. The complete plant transformation cassette containing fused gene fragments of *aquaporin* (*AQP*) and *alpha glucosidase* (*AGLU*) in sense and anti-sense orientation with an intron in between to facilitate hairpin structure formation was cloned in pCambia2300 binary vector between *Sac*I and *EcoR*V sites.

Sequences of two target genes (*AQP* and *AGLU*) expressed in the whitefly midgut and involved in osmoregulation were selected (Brown lab database UA, USA). Partial fragments from both the genes (200bp each) were joined in sense and antisense orientation separated by an intron and synthesized commercially (Life technologies, Thermo Fisher Scientific, USA). The desired restriction sites were also introduced into the construct for initial as well as final cloning in desired vector system. The synthesized fragment (931bp) was initially cloned into a pJIT163 vector [35] in order to express mRNA as a hairpin structure for dsRNA generation under the control of the *Figwort mosaic virus* (FMV) promoter and CaMV35S terminator. The 2xCaMV35S promoter already present in the pJIT163 vector was replaced by FMV promoter using *Sac*I and *Hind*III restriction sites. The complete cassette including the FMV promoter, synthesized construct and CaMV35S terminator was restricted from pJIT163 using *Sac*I and *EcoR*V restriction sites and cloned into the binary vector pCambia2300 (Fig 1;GSL Biotech LLC Chicago, USA) which was subsequently used for plant transformations. The *EcoR*V restriction site produced a blunt end cut and was used because of the limited number of options available for further cloning. The pCambia2300 vector was restricted using *Sac*I and *Sma*I sites as *Sma*I also produced a blunt end and the cassette was ligated.

### Development of transgenic plants

**Fig 2:**
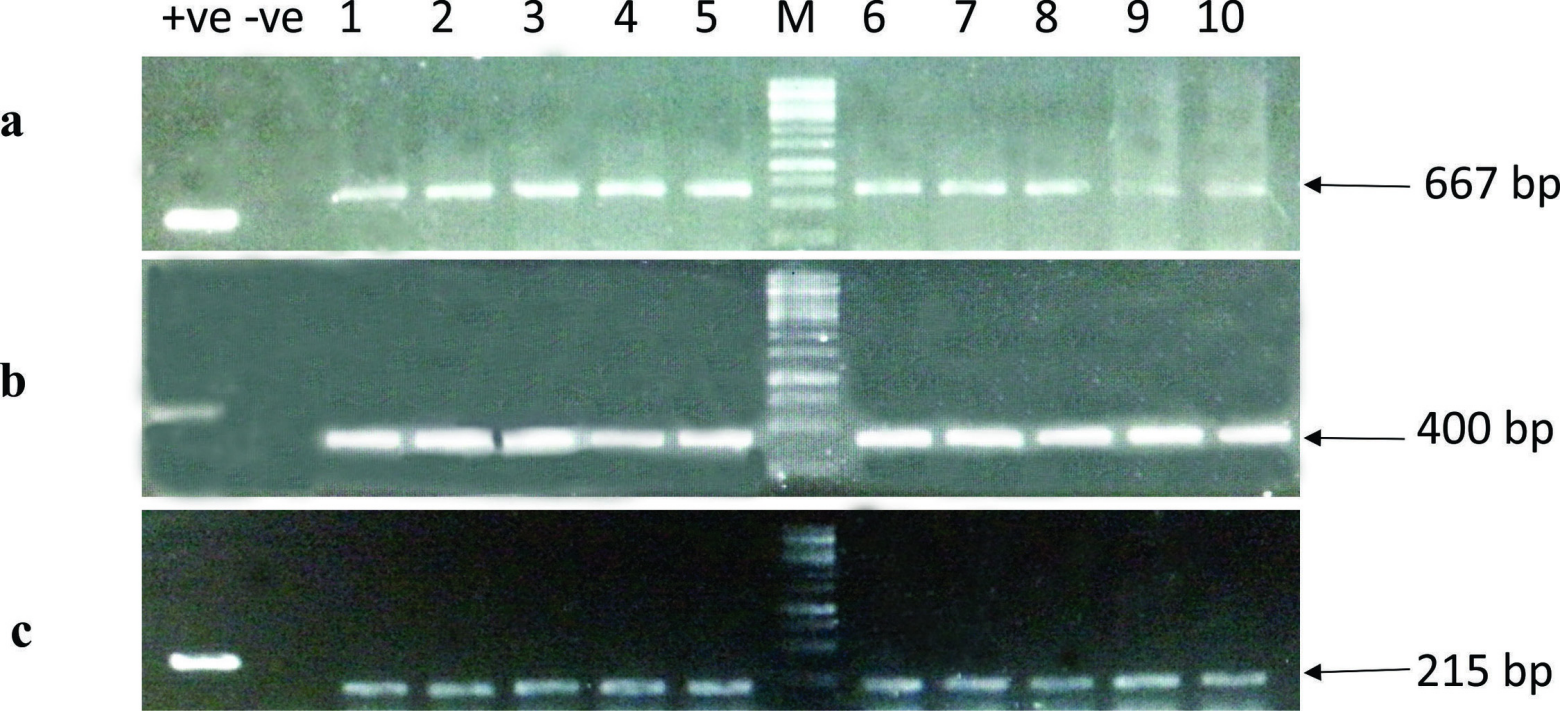
PCR confirmation of transgenic plants. PCR amplification of (a) FMV promoter, (b) gene of interest and (c) *nptII* gene. +ve = Positive control, -ve = Negative control, Lane 1–5 = G 1.2, M = Marker, Lane 6–10 = G 1.3

The final construct containing partial fragments of the two genes for the expression in transgenic plants was developed in the pCambia2300 vector system. The complete cassette cloned into the plant transformation binary vector pCambia2300 was transformed into *Agrobacterium tumefaciens* LBA4404 strain using an electroporation method. Transgenic plants with the double gene construct were developed by a modified direct organogenesis method using leaves as explants for the *Agrobacterium*-mediated plant transformation [36]. Kanamycin selection at a concentration of 500 mg/L was used in order to identify the transformants. Transgenic plants were grown in tissue culture growth rooms maintained at a controlled temperature of 25±2°C and later transferred to plastic pots and moved to the glasshouse for seed setting and bioassays. The glasshouse was also maintained at 25±2°C with a relative humidity of 60–70%. Total DNA from transgenic plants was isolated and used as template for confirmation through PCR (Fig 2). The PCR reaction was carried out using Dream Taq Green PCR Master Mix (2x) (Thermo Fisher scientific, USA) and cycling parameters were 94°C for 3 min (1 cycle), 94°C for 30 sec, 54°C for 30 sec and 72°C for 45 sec (35 cycles), with a final extension cycle at 72°C for 10 min.

### Insect bioassays

A total of five transgenic lines were generated initially and after confirmation through PCR, two transgenic lines G1.2 and G1.3 were selected for insect bioassays. For each transgenic line, twenty plants were generated and insect mortality was evaluated by comparing transgenic lines with mock (positive control) and non-transgenic (negative control) plants. Transgenic as well as control plants were grouped in separate cages (five plants per group). For each replicate around 250 newly hatched whiteflies were transferred to each group of plants by gently tapping the plants containing whiteflies in order to avoid any mechanical damage to the insects, which could give erroneous mortality data. Initial count of the insects was recorded once they were settled on the walls of the glass cages (any additional insects were removed using aspirator) and plants were then placed in the cages for bioassays. The experiment was repeated twice and mortality was recorded on daily basis for six consecutive days. The means for percent mortality were calculated and data were analyzed statistically for each set of plants.

### Statistical analysis

The experimental data were statistically analyzed by one way analysis of variance (One-way ANOVA) followed by Tukey’s multiple comparison test of significance using Graph Pad Prism 5 software.

### RNA extraction and cDNA synthesis

Transgenic lines as well as *N*. *tabacum* control plants were exposed to adult whiteflies and then insects were collected from each group of plants after 1, 3 and 6 days post feeding. Total RNA from whiteflies (25 whiteflies were collected for RNA isolation from each group of plants)collected at each time point from transgenic and/or control plants was isolated using TRIzol^®^ Reagent (Thermo Fisher Scientific, USA) and was treated with DNaseI as per manufacturer’s recommendations (Thermo Fischer scientific, USA). The RNA samples were quantified using a Nanodrop 2000 spectrophotometer (Thermo Fischer scientific, USA), and 0.5 μg DNase treated RNA was processed for first strand cDNA synthesis as per manufacturer’s instructions using Maxima First Strand cDNA Synthesis Kit for RT-qPCR (Thermo Fisher Scientific, USA).

### Real-time quantitative PCR analysis

Real time quantitative PCR (RT-qPCR) was performed to measure relative expression of the genes *AQP* and *AGLU* in *B*. *tabaci*. A total volume of 25μl reaction mixture contained 12.5μl SYBR^®^ Green Real-Time PCR Master Mix (Thermo Fisher Scientific, USA), 0.25μl each of the forward and reverse primers (Table 1;0.1 pmole), 2.5μl cDNA (~25 ng) and 9.5μl water. After optimization, the thermocycling conditions were: 1 cycle at 94°C for 10 min followed by 40 cycles, at 94°C (for 30 sec.), 57°C (for 30 sec.) and 72°C (for 30 sec.). The RT-qPCR was performed in a 96 well microtiter plate using Bio-Rad iQ5 thermal cycler (Bio-Rad, USA). Full experiments with all necessary controls were triplicated. At the end of every run, a melt curve analysis was performed from 60 to 95°C, with an increment of 0.5°C every 10 sec. in order to assess the specificity of the amplification product. Quantification results were analyzed by ΔΔCt method [37] and the 18S ribosomal RNA (rRNA) gene (GenBank accession number Z15051.1) was used to normalize the corresponding Ct values. Transcript levels were measured in whiteflies fed on transgenic plants and/or control plants and the fold change in the expression levels of *AQP* and *AGLU* were determined.

**Table 1:** Primer pairs used for the assessment of expression down regulation of target genes of *Bemisia tabaci* using RT-qPCR.

Gene	Forward primer	Reverse primer
**Aquaporin**	qAQPF5’-CCTGCATTCGTCAGTGGAATTTG-3’	qAQPR5’- GCAGTGACTCCACCGAGTATT-3’
**Alpha glucosidase**	qAGLUF5’-CACCGCGTCGAACCTCATG-3’	qAGLUR5’- GCGAAGAGTTGGTTCAAGAGATG-3’
**18S rRNA**	q18SF5’-GACCGGAGCTTGCAATTGTTC-3’	q18SR5’- ATCGCCGCGAGGTTATGAC-3’

## Results

### Development of construct for expressing dsRNA targeting osmoregulators of whitefly in transgenic tobacco plants

Two target genes *AQP* and *AGLU* were selected for studies to investigate whether the silencing effect of these endogenous genes could affect physiological processes in whitefly. The construct was designed in such a way that the short fragments (200 bp) of both the targets were fused under the control of strong universal promoter (FMV promoter) in order to express siRNAs simultaneously against both the genes. For the assessment of plant resistance to whitefly, transgenic *Nicotiana tabacum* plants were generated using the construct expressing hairpin loop mRNA inserted into plant expression vector pCambia2300. Total DNA was isolated from transgenic plants and insertion of T-DNA was confirmed by PCR (Fig 2) using primers specific for the gene as well as the FMV promoter and *nptII* gene (Table 2).

**Table 2:** Primer pairs used for the confirmation of transgenic plants through PCR.

Gene	Forward primer	Reverse primer
**FMV promoter**	FMVF5’-GAGCTCCAAGTGTACAAAAAAGATTACCA-3’	FMVR5’-AAGCTTGCACGCCGTGGAAACAGAAGACA-3’
**Gene of interest**	G1F5’-GAGCCATCTGTGGAGCAATCATT-3’	G1R5’-CAGGGAGGTCTTCGATTTCTACA-3’
**nptII gene**	npt5-‘ CTCACCTTGCTCCTGCCGAGA-3’	npt5’- CGCCTTGAGCCTGGCGAACAG-3’

### Expression down regulation analysis of *B*. *tabaci*

Insect feeding on plants expressing dsRNA against genes involved in osmoregulation can reduce the transcript level of the target genes and cause physiological alterations leading to osmoregulatory disturbances in the insect gut and early mortality. In this study, two genes were selected and fused for the development of an RNAi construct which was then transferred into the tobacco plants (*Nicotiana tabacum*) in order to produce long dsRNA in transgenic plants that will ultimately be processed into siRNAs. The dsRNA in transgenic tobacco plants was expressed under the control of a strong constitutive FMV promoter which will also results in higher level expression in companion cells. Real time qPCR was performed to assess the suppression of gene expression in *B*. *tabaci*. Ingestion of sap from transgenic tobacco plants producing dsRNA corresponding to *AQP* and *AGLU* was shown to decrease the expression of both genes in *B*. *tabaci*. Gene expression profiling using RT-qPCR was performed for both the genes after exposing whiteflies to transgenic plants and the results were compared to the controls.

**Fig 3:**
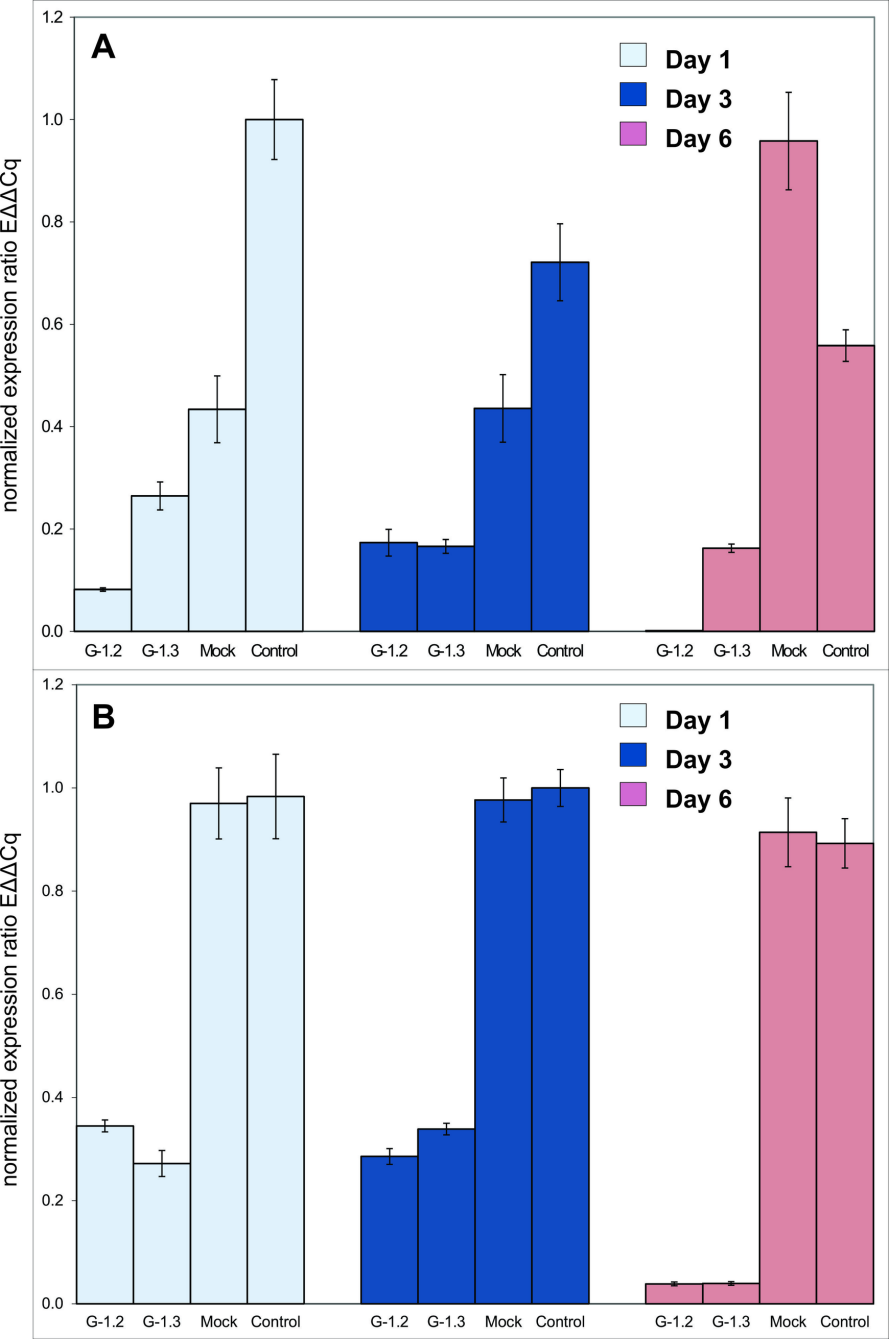
Expression profiling using real time qPCR of genes expressed in the midgut of *B*. *tabaci*. Real time qPCR results demonstrate fold change in gene expression in terms of significant level of gene expression knock down after one, three and six days of feeding whiteflies on transgenic plants compared to the whiteflies fed on control plants. (A) Shows the difference in gene expression of *aquaporin* compared to the controls while (B) demonstrates the comparison of expression level of *alpha glucosidase*.

The data obtained from the RT-qPCR demonstrated significant reduction in the expression levels of both the genes in whiteflies fed on transgenic plants, compared to the whiteflies fed on control plants over a period of six days (Fig 3). The whiteflies were collected from transgenic as well as control plants at three different time points for expression down regulation assessment. In case of *AQP* around 90% reduction in mRNA expression was recorded after 24 h of feeding, especially in case of whiteflies feeding on transgenic line G1.2 whereas the reduction in expression was around 75% in whiteflies feeding on transgenic line G1.3. The level of gene expression remained at ~ 80% suppression level in whiteflies collected after three days of feeding from both the transgenic lines compared to the control. The expression level of *AQP* was almost undetectable after six days of feeding on G1.2 compared to G1.3 where the suppression level around 80% compared to the controls was observed (Fig 3A). *Alpha glucosidase* mRNA seems to be less responsive to the RNAi treatment compared to the *aquaporin* initially, and both the transgenic lines behaved in a similar fashion against *AGLU*. The expression level of *AGLU* in whiteflies fed on both transgenic lines was suppressed by 60% compared to the controls after 24 h of feeding and remained so, even after three days of feeding. The whiteflies collected after six days of feeding demonstrated that the expression level was further repressed to more than 90% in comparison to the controls (Fig 3B).

### Disruption of osmoregulation caused early mortality

**Fig 4:**
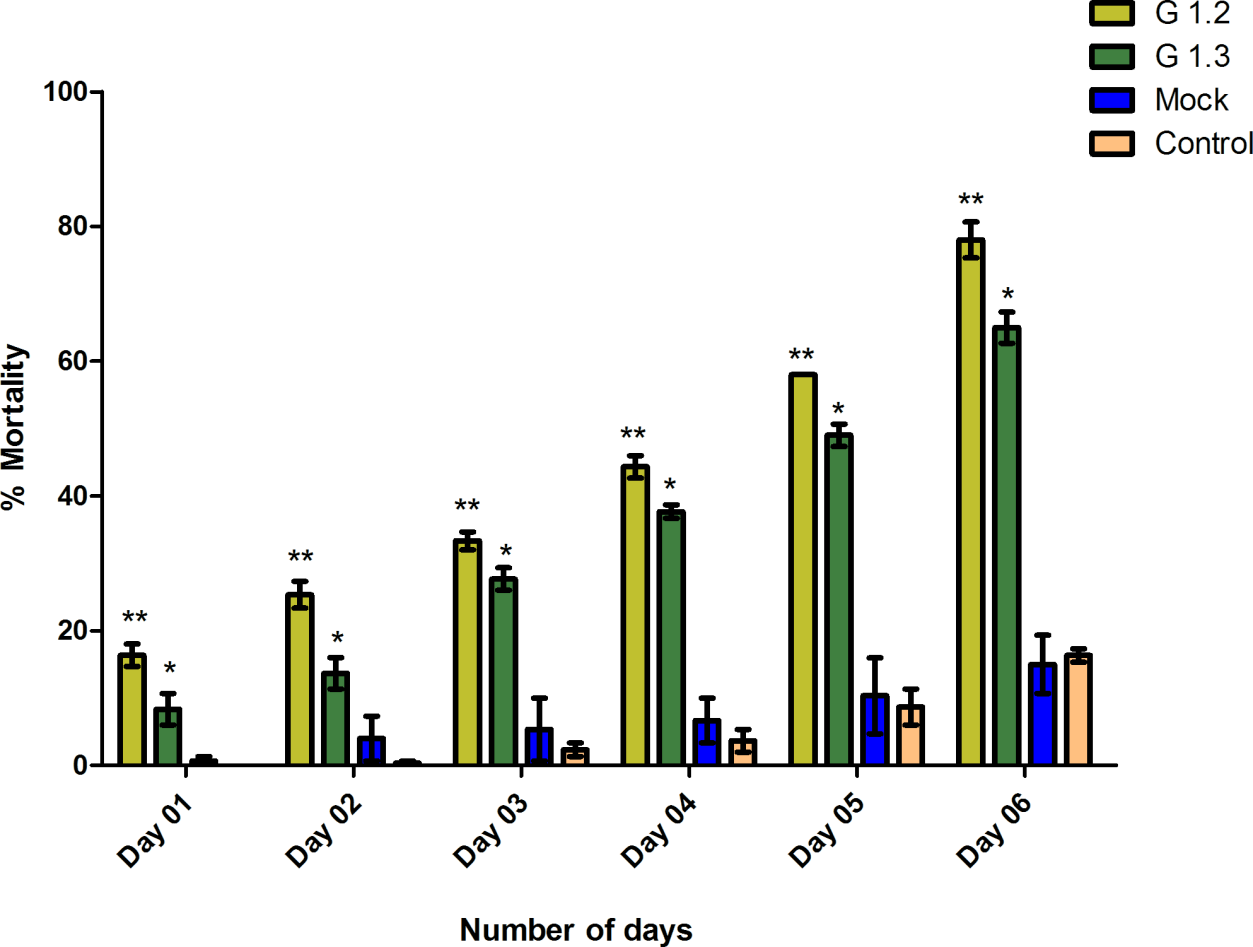
Statistical analysis of mean percent mortality. Mortality results were statistically analyzed and the bar chart shows comparison of mortality between transgenic lines being compared to the mock and control plants (G1.2 & G1.3 represents two different transgenic lines). *p<0.05, **p<0.01.

The survival of *B*. *tabaci* depends on the ability to maintain osmotic pressure inside the body. Expression down regulation of both *AQP* and *AGLU* caused significant mean percent mortality over a period of six days with 78% and 65% for transgenic lines G1.2 and G1.3 respectively compared to the controls (around 16% mortality in both the controls) after six days of feeding. RNAi mediated disruption of function of these genes crucially affected the survival rate of the insect. Mean percent mortality in the whiteflies feeding on transgenic plants compared to the controls was recorded and the results were analyzed statistically (Fig 4).

## Discussion

Whitefly management is extremely difficult because of its short life cycle and high reproductive rate. A number of studies demonstrated proof of concept for the control of crop pests through gene silencing using RNAi approach. Delivery of dsRNA to the insect’s midgut using a transgenic plant mediated RNAi approach can cause severe physiological problems including defective molting and osmoregulatory disruption [26, 32]. Osmoregulation is the key to insect survival particularly for sucking insect pests. *Alpha glucosidases* have also been reported to facilitate viruses by playing important role in proper folding of glycoproteins involved in encapsidation of viral genetic material, and the inhibitors of alpha glucosidases were shown to act as anti-viral agents [38]. Observed pathogenicity of transgenic plants expressing dsRNA as *AGLU* inhibitor were symptomless compared to the control plants which showed typical begomovirus infection symptoms, but the phenomenon needs to be explored by under taking relevant experiments. Structural analysis in a study conducted on aphids, another hemipteran insect, demonstrated that the *AQP* contains all the important amino acids required for selective and efficient water transport [27]. RNAi has been used successfully for reverse genetics studies although, the susceptibility of insect species towards RNAi varies [22, 23, 39]. Preliminary studies carried out in *B*. *tabaci* using artificial diet based feeding of dsRNA corresponding to different important genes demonstrated considerable variation in susceptibility of target genes towards dsRNA mediated suppression (our unpublished data). Although, the dsRNA uptake machinery is present in *B*. *tabaci* [40], there may be other factors required for some genes to be silenced. In the present study, the combined fragments of two important genes were used in a single construct provided suppression of whitefly. In this study, the hairpins were successfully inserted and their concentration in planta was sufficient to induce a strong RNAi response against the selected mRNA targets in whitefly, leading to significantly increased mortality. This strategy will ensure the continuous production and availability of dsRNA in the sieve elements where they can move to more distant plant parts through the phloem [41].

Variation in dsRNA expression can occur depending on the integration site in the plant genome. It is also essential that the integration of any foreign gene should not disturb the morphology or physiology of the plant as in this study, where no such variations were observed in the transgenic tobacco plants. Although this study successfully used the constitutively expressed FMV promoter, it may also be possible to use a tissue specific or insect bite inducible promoter [42] to more precisely target the expression of the genes. Plant mediated resistance using RNAi-based approaches has also been achieved against nematodes and bacterial as well as viral diseases [43–46]. Another study recently demonstrated that the expression of long dsRNA in plastids (chloroplast) can result in complete plant protection against beetles [47], which may offer additional options for resistance against plant pests. Selection of the target gene is of utmost importance for the effectiveness of RNAi. Although the RNAi knockdown experiments carried out on *C*. *elegans* demonstrated lethal phenotypes, there is no universal set of target genes available for broad-spectrum protection against nematodes, indicating the effect is highly species specific [48]. This is also true for various insect species of agricultural importance and careful selection of the target gene(s) for a particular insect species will be required. It will also be important to minimize the effects on beneficial or non-target insects that may feed on the transgenic plants in order to achieve a fully successful transgenic plant mediated resistance strategy [22, 45, 49]. This study adds more evidence for the potential uses of emerging dsRNA technologies using combinations of genes and will open up new avenues for gene pyramiding. RNAi can also be complemented with other approaches such as Bt technology for broader spectrum resistance against sucking as well as chewing insects. It will also be interesting to determine the homology of the gene targets among different *B*. *tabaci* species as well as with the genes of other important insect pests belonging to different orders for the development of one single RNAi based strategy for broad spectrum control of insect pests.
